# The PI3K/Akt/mTOR Signaling Pathway in Triple-Negative Breast Cancer: A Resistance Pathway and a Prime Target for Targeted Therapies

**DOI:** 10.3390/cancers17132232

**Published:** 2025-07-03

**Authors:** Ali Hassan, Corinne Aubel

**Affiliations:** 1 INSERM, U1240 IMoST, Imagerie Moléculaire et Stratégies Théranostiques, Université Clermont Auvergne, 63000 Clermont-Ferrand, France; 2 Département d’Oncogénétique, Laboratoire d’Oncologie Moléculaire, Centre Jean Perrin, 58 rue Montalembert, 63011 Clermont-Ferrand, France

**Keywords:** triple-negative breast cancer (TNBC), PI3K, AKT, mTOR, targeted therapies

## Abstract

Triple-negative breast cancer is the most aggressive subtype of breast cancer and is associated with the worst prognosis. Conventional chemotherapy remains the gold standard treatment for this disease but is associated with a high relapse rate, highlighting the urgent need for effective targeted therapies. The PI3K/Akt/mTOR pathway, dysregulated in nearly 60% of these cancers, appears to be a prime target. It involves a signaling cascade beginning with PI3K activation followed by activating phosphorylation of Akt and then mTOR complex, which activates oncogenic processes by enhancing protein synthesis, inhibiting apoptosis, dysregulating autophagy and promoting DNA repair that supports tumor cell survival. Moreover, the PI3K/Akt/mTOR pathway plays a central role in the development of chemoresistance. Numerous alterations (activating the mutation of PIK3CA or the loss of tumor suppressor PTEN) may lead to its overactivation. Targeted inhibitors of PI3K, Akt and mTOR have been developed to counteract this dysregulation. However, numerous cancer resistance mechanisms have emerged, reducing their efficacy, for example, reactivation of Akt following mTOR blockade, reactivation of the pathway by insulin signaling or activation of compensatory pathways such as the MAPK pathway, thus limiting their integration into routine practice. To counteract these resistances, combination therapies currently being investigated in clinical trials aim to improve clinical outcomes of PI3K/Akt/mTOR pathway inhibition. The aim of this review was to summarize current therapies developed to target this pathway in TNBC, with a focus on the resistance mechanisms that limit their effectiveness.

## Simple Summary

The PI3K/Akt/mTOR pathway is a key cell signaling pathway involved in many processes involved in carcinogenesis, such as proliferation, cell migration and inhibition of apoptosis. Due to activating mutations in PIK3CA and AKT1, and loss-of-function mutations in the tumor suppressor PTEN, it is frequently hyperactivated in triple-negative breast cancer, making this subtype of breast cancer particularly aggressive and resistant to conventional therapies. Several inhibitors of this pathway have been developed, but numerous molecular resistance mechanisms limit their clinical efficacy. To overcome these resistances, therapeutic approaches combining PI3K/Akt/mTOR inhibitors with other targeted therapies are currently under investigation. In the era of personalized medicine, this approach could improve the prognosis of patients with this disease.

## 1. Introduction

Triple-negative breast cancer (TNBC) accounts for approximately 20% of all breast cancers (BC) [1]. Its phenotype is characterized by a lack of estrogen receptor (ER), progesterone receptor (PR) and absence of Human Epidermal Growth Factor Receptor 2 (HER2) overexpression [2]. This cancer subtype is more frequent in women under 40 years old [3]. TNBC is highly heterogeneous and has been traditionally classified into six subtypes by Lehmann et al.: Basal-like 1 and 2 (BL1 and BL2), Immunomodulatory (IM), Mesenchymal (M), Mesenchymal Stem-like (MSL) and Luminal Androgen Receptor (LAR) [4]. In 2016, this classification was revised to retain four subtypes: BL1, BL2, M and LAR [5]. Moreover, it has high proliferation and invasion rates [6], which explains why it is the most aggressive subtype of BC and is associated with worse prognosis [7].

Its specific profile (ER-/PR-/HER2-) makes it ineligible for endocrine and anti-HER2 therapies [1]. To date, conventional chemotherapies such as anthracyclines or taxanes [8] remain the first-line neoadjuvant treatments. However, these therapies are associated with a high risk of relapse and significant toxicity, which limits their clinical efficacy [9]. It, therefore, seems urgent to identify potential therapeutic targets against this disease, particularly in the era of personalized precision medicine.

Therefore, the PI3K/Akt/mTOR pathway, activated in around 60% of TNBC [10], has been a prime target for therapeutic development. PI3K/Akt/mTOR interacts with numerous other cellular signaling pathways, and its deregulation is associated with the development and progression of many types of cancer [11].

The aim of this review was to provide a comprehensive overview of PI3K/Akt/mTOR-targeted therapies in TNBC, along with the resistance mechanisms affecting their efficacy.

## 2. The PI3K/Akt/mTOR Signaling Pathway in TNBC

**Figure 1:**
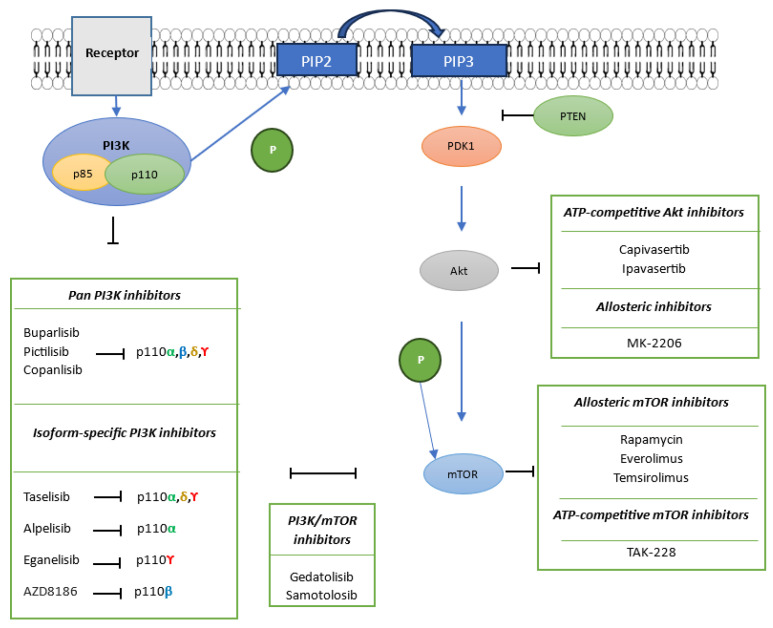
PI3K/Akt/mTOR signaling pathway and the respective inhibitors used in TNBC. The corresponding receptor activates PI3K in dimerized form (p85 and p110 subunits). Activated PI3K phosphorylates PIP2 to PIP3, activating the Akt signaling cascade, which in turn phosphorylates mTOR. PTEN is the main natural inhibitor of this signaling pathway, acting upstream of PDK1. PI3K inhibitors are represented by pan-pi3k inhibitors (buparlisib, pictilisib and copanlisib) and isoform-specific PI3K inhibitors (taselisib, alpelisib and eganelisib). Akt inhibitors are divided between ATP-competitive inhibitors (capivasetib and ipavasertib) and allosteric inhibitors (MK-2206). mTOR inhibitors are divided between allosteric inhibitors (rapamycin, everolimus and temsirolimus) and ATP-competitive inhibitors (TAK-228). Finally, PI3K/mTOR inhibitor are represented by gedatolisib and samotolisib.

PI3K is a heterodimeric lipid kinase composed of a p110 catalytic subunit, including isoforms α (encoded by PIK3CA), β, δ and ϒ, and the p85 regulatory subunit that contains an N-terminal Src homology 2 (SH2) domain, which binds to receptor tyrosine kinases (RTK) to activate PI3K (Figure 1) [12]. Activated PI3K catalyzes phosphorylation of phosphoinositide-4,5-biphosphate (PIP2) to phosphoinositide-3,4,5-triphosphate (PIP3), which recruits phosphoinositide-dependent kinase 1 (PDK1) and Akt to the plasma membrane by interacting with their plekstrin homology (PH) domains [13]. The main natural inhibitor of this step is the tumor suppressor Phosphatase and TENsin homolog (PTEN) [14], which dephosphorylates PIP3 back to PIP2 through its inositol polyphosphate 3-phosphatase activity, decreasing Akt phosphorylation [15].

At the plasma membrane, Akt is phosphorylated on threonine 308 of its kinase domain by PDK1, and at serine 473 within its regulatory domain by mTORC2, resulting in full activation. Activated Akt subsequently phosphorylates its downstream targets [16,17] (Figure 1), including the mTORC1 and mTORC2 protein complexes, of which the mTOR serine/threonine kinase is the catalytic subunit [18].

## 3. The PI3K/Akt/mTOR Pathway in Oncogenesis and Resistance to Anti-Cancer Therapies in TNBC

The PI3K/Akt/mTOR pathway regulates several essential physiological cellular processes, including survival, growth and migration [19].

### 3.1. Role in Protein Translation

Once activated, mTORC1 plays a central role in translation initiation by phosphorylating its two main substrates: ribosomal S6 kinase 1 (S6K1) and eukaryotic translation initiation factor 4 E-binding protein 1 (4E-BP1) [20]. In fact, phosphorylation of 4E-BP1 leads to the release of eukaryotic translation initiation factor 4E (eIF4E), which then initiates protein translation at the 5′ end of mRNAs [21]. In addition, S6K1 phosphorylation promotes the assembly of the translation initiation complex [20,22] by phosphorylation of ribosomal protein S6 and eIF4B [23].

### 3.2. Role in Epithelial Mesenchymal Translation

Epithelial–mesenchymal transition (EMT) is a process by which an adherent epithelial polar cell acquires a mesenchymal phenotype. This process, essential for cell migration during embryogenesis and organogenesis, is strongly implicated in cancer metastasis [24]. EMT is enhanced by the PI3K/Akt/mTOR pathway. In fact, mTORC1/eIF4E axis enables protein translation, and mTORC2 acts at the post-translational level by stabilizing Snail [25]. Also, PTEN loss decreases cell polarity and commits cells to EMT [26].

### 3.3. Role in Apoptosis

The PI3K/Akt/mTOR pathway play also a central role in apoptosis inhibition. Indeed, the anti-apoptotic proteins B-cell lymphoma 2 (Bcl-2) and X-linked inhibitor of apoptosis protein (XIAP) are frequently overexpressed in PIK3CA-mutated TNBC cells [27]. In addition, the eIF4F complex activated by mTORC1 promotes Myeloid Cell Leukemia-1 (MCL-1) anti-apoptotic protein translation [28]. Akt downregulated apoptosis through inhibitory phosphorylation of the pro-apoptotic protein bcl-2 antagonist of cell death (BAD) [29] and Forkhead box O (FoxO) transcription factors 3 and 1, which are involved in the regulation of apoptosis [30,31].

### 3.4. Role in Autophagy Regulation

Moreover, mTORC1 inhibits autophagy by phosphorylating key autophagy markers such as autophagy-related protein 13 (ATG13) and Unc-51-like autophagy activating kinase 1 (ULK1) [32] and by promoting the cytoplasmic retention of the lysosomal gene expression modulator Transcription Factor EB (TFEB), thereby enhancing cell survival [33].

### 3.5. Role in Cell Morphogenesis

The other subunit of the mTOR complex, mTORC2, also contributes to tumor development. It promotes cell morphogenesis and migration through phosphorylation of protein kinase C δ and α [34,35], which regulates actin cytoskeleton dynamics [33]. In addition, PTEN also exerts a non-phosphatase activity that contributes to chromosome stability and enables it to act as a scaffolding protein in the nucleus and the cytoplasm [36].

### 3.6. Role in DNA Repair

Independently of mTOR, PI3K and Akt play fundamental roles in genome stability and DNA repair. Notably, interactions between PI3K and homologous recombination (HR) are essential for the repair of DNA double-strand breaks [37]. Furthermore, Akt induces the degradation of the transcription factor Forkhead box (Fox) O3 and FoxO1, thereby enabling the expression of FoxM1 and exonuclease 1, which regulate the expression of Breast Cancer 1 (BRCA1), BRCA2 and RAD 51, key components of the HR system [38]. Activation of the HR system repairs DNA double-strand breaks. This increases cell survival and generates drug resistance by maintaining the genetic integrity of cells damaged by treatment [37].

### 3.7. Role in Chemoresistance

In addition to its established role in oncogenesis, the PI3K/Akt/mTOR pathway is strongly implicated in chemoresistance. Several commonly used chemotherapies in TNBC promote Akt phosphorylation, thereby altering drug response [39]. Once activated, Akt enhances the activation of nuclear factor erythroid 2-related factor 2 (Nrf2), which subsequently promotes Multidrug-Resistant (MDR) protein expression, leading to drug resistance through cellular efflux [40,41].

Moreover, PI3K/Akt/mTOR can lead to immune evasion. In fact, PTEN loss has been associated with resistance to anti-PD-1/PD-L1 therapies by impairing T-cell CD8+ infiltration and promoting angiogenesis through vascular endothelial growth factor (VEGF) overproduction [42].

These chemoresistance mechanisms result in widely variable drug responses. For example, the pathologic complete response (pCR) rate reaches 52% for the BL1 subtype but drops to just 10% for LAR subtype and 0% for BL2 tumors [43,44].

## 4. PI3K/Akt/mTOR Pathway Alterations in TNBC

The main alterations of the PI3K/Akt/mTOR pathway involve activating mutations in PI3K and downregulation of PTEN, both of which drive hyperactivation of the pathway [40]. In TNBC, PIK3CA mutations occur in 10.2% of cases, with a higher frequency in the LAR subtype (46.2%) compared to other subtypes (4.5%) [45]. Approximately 80% of PIK3CA somatic mutations involve three hotspot mutations, each affecting a single amino acid. E542K and E545K, located in exon 9 (helicase domain), disrupt interactions with the SH2 domain of the p85 regulatory subunit. H1047R, in exon 20 (kinase domain), upregulates activation of downstream PI3K signaling proteins [27]. Mutations in the p85α subunit are less frequent but can also activate the PI3K pathway. Additionally, PIK3CA amplification can result in allelic dose-dependent activation of the pathway [46].

A reduction in PTEN expression of 20% may be sufficient for the development of mammary tumors. PTEN expression is decreased in 19% of BCs and in up to 35% of TNBCs [47,48], particularly in BL subtypes [9]. Loss of PTEN, and, therefore, of its 3′-phosphatase activity, leads to an increase in PIP3, constitutively activating the PI3K pathway, which promotes cell proliferation and tumor progression [49]. PTEN loss can be caused by allelic inactivation leading to loss of function and homo- or heterozygous deletions. Promoter hypermethylation can also silence PTEN expression at the transcriptional level. Furthermore, post-transcriptional modifications, such as microRNA-498 overexpression, are linked to decreased PTEN expression and function. Finally, PTEN protein can undergo various post-translational modifications, such as phosphorylation, acetylation, oxidation and ubiquitination, which can alter its activity [50].

The Akt1 E17K hotspot mutation is the most common Akt-related alteration, promoting Akt1 binding to PIP3 and stimulating its activation [33]. Although Akt1 mutations are uncommon (3%) [51], they are more frequently observed in TNBC expressing androgen receptors [44].

## 5. PI3K/Akt/mTOR Pathway Targeted Therapies for TNBC

The central role of the PI3K/Akt/mTOR pathway in tumorigenesis has led to the development of numerous targeted therapies, some of which are used in TNBC.

### 5.1. PI3K Inhibitors in TNBC

PI3K inhibitors are divided into pan-PI3K inhibitors, targeting all isoforms of the p110 subunit and isoform-specific inhibitors [52] (Figure 1).

Among PI3K pan-inhibitors, copanlisib has demonstrated anti-proliferative effects in TNBC cell lines SUM149 and BCX010, reducing colony formation to 40% and 50% of control levels, respectively, in preclinical assays [53]. However, this drug has not been evaluated as monotherapy in TNBC in clinical trials. Pictilisib (GDC-0941) did not show efficacy in TNBC cell lines MDA-MB-231 and HCC1937 in preclinical studies, with percentage cell survival rate exceeding 90% [54]. To date, the most extensively studied pan-PI3K inhibitor in TNBC is buparlisib (BKM120). Its use as monotherapy in patients with metastatic disease was assessed in the phase II clinical trial NCT01790932 (Table 1). The median progression-free survival (PFS) of the fifty patients enrolled was only 1.8 months (95% confidence interval (CI): 1.6–2.3), and the median overall survival (OS) was 11.2 months (95% CI: 6.2–25). Fatigue was the most frequent adverse event (58% all grades, 8% grade 3), followed by nausea (34% all grades, none grade 3) [55]. Furthermore, the randomized phase II/III study BELLE-4 (NCT01572727), comparing the use of buparlisib versus placebo in combination with paclitaxel, notably in a TNBC cohort (*n* = 90), showed a median PFS in the buparlisib plus paclitaxel arm of 5.5 months (95% CI: 3.5–7.3) compared to 9.3 months (95% CI: 3.7-not evaluable) in the placebo plus paclitaxel arm, indicating limited efficacy in advanced TNBC [56].

Several isoform-specific PI3K inhibitors have also been developed and evaluated in TNBC (Figure 1) to improve selectivity and reduce off-target toxicity. The PI3Kβ inhibitor AZD8186 demonstrated good antitumor efficacy in PTEN-deficient TNBC cell lines BT549, MDA-MB-468 and MDA-MB-436, with half-maximal inhibitory concentrations of 31, 358, and 899 nM, respectively [57].

The NCT01884285 phase I trial confirmed a good safety profile of AZD8186 for future clinical development in a cohort of 161 patients, including TNBC cases, with only four patients experiencing dose-limiting toxicities [58]. Alpelisib, a PI3Kα inhibitor, showed conversely a poor tolerability profile. In fact, in the phase II clinical trial NCT02506556 involving 43 patients with advanced breast cancer (including 10 TNBC patients), 62.7% of patients experienced adverse events of at least grade 3. The most frequent adverse events were hyperglycemia (32.6%), maculopapular rash (25.6%) and colitis (7%) [59]. Similarly, taselisib, targeting PI3Kα, δ and ϒ isoforms, was also poorly tolerated in Phase I clinical trial NCT01296555. In fact, the most common adverse events were diarrhea (57.8%), followed by nausea (39.8%), fatigue (32.5%), decreased appetite (31.9%), and hyperglycemia (31.3%). Furthermore, 66.9% of all patients experienced at least one grade ≥ 3 adverse event [60].

Concerning clinical outcomes, alpelisib, showed disappointing results in the trial NCT02506556, which included 10 TNBC patients [59]. For these patients, the median PFS in intention-to-treat cohort was only 1.8 months (95% CI: 1.7—not evaluable), and the median OS was 5.3 months (95% CI: 2.9—not evaluable). Similarly, taselisib, assessed as monotherapy in the phase I clinical trial NCT01296555 in patients with *PIK3CA*-mutant cancers [60], showed a limited benefit in TNBC cohort (*n* = 17; 10.2%), with a median PFS of 3.0 months (95% CI: 1.6–4.2).

Last, the PI3K-ϒ inhibitor eganelisib (IPI-549) showed no antitumor effect as monotherapy (except for one patient with peritoneal mesothelioma) in the MARIO-1 phase I/Ib clinical trial (NCT02637531) (Table 1) [61].

### 5.2. Akt Inhibitors in TNBC

Akt inhibitors are classified into ATP-competitive and allosteric inhibitors and are being developed to overcome TNBC chemoresistance (Figure 1) [62].

Capivasertib and ipatasertib are among the ATP-competitive inhibitors [63,64,65]. Capivasertib was compared with placebo, both in association with paclitaxel, in the phase II trial PAKT (NCT02423603), assessing efficacy in women with untreated metastatic TNBC. The median PFS was 5.9 months (95% CI: 3.8–7.5) in the paclitaxel plus capivasertib arm, compared to 4.2 months (95% CI: 3.5–5.2) in the paclitaxel plus placebo arm. More interestingly, the addition of capivasertib in the PIK3CA/Akt/PTEN-altered subgroup led to a median PFS of 9.3 months (95% CI: 3.7–17.7) versus 3.7 months (95% CI: 1.9–5.9) in the paclitaxel plus placebo arm (Table 1) [63]. For its part, ipatasertib efficacy in association with paclitaxel was evaluated in the phase II trials LOTUS (NCT02162719) (metastatic TNBC) [64] and FAIRLANE (NCT02301988) (early TNBC) [65]. In the LOTUS trial, including 62 patients in each arm, the median PFS was longer in the paclitaxel plus ipatasertib arm (6.2 months; 95% CI: 3.8–9.0) than in the paclitaxel plus placebo arm (4.9 months; 95% CI: 3.6–5.4). This benefit was more pronounced in the PTEN-low patients, where median PFS was 6.2 months (95% CI: 3.6–9.1) in the paclitaxel plus ipatasertib arm, compared to 3.7 months (95% CI: 1.9–7.3) in the paclitaxel plus placebo arm. In the FAIRLANE trial, in which 76 patients were included in the paclitaxel plus ipatasertib bi-therapy arm and 75 patients in the paclitaxel plus placebo arm, the authors concluded that ipatasertib did not improve the pathologic complete response (pCR) (17.1% versus 13.3%), but it did improve the overall response rate by 11.1% (95% CI: 5.6–27.9) in the intention-to-treat population, by 23.7% (95% CI: 13.6–60.9) in the PTEN-low population and by 14.9% (95% CI 12.4–42.3) in the PIK3CA/Akt1/PTEN-altered population. These data suggest that capivasertib and ipavasertib could improve disease prognosis in first-line therapy when used in combination with paclitaxel, particularly for TNBC with PI3K/Akt/PTEN alterations.

Among the allosteric inhibitors (Figure 1), MK-2206 has been evaluated as monotherapy in the phase II trial NCT01277757 in patients with advanced breast cancer and tumors harboring PIK3CA or Akt mutations and/or PTEN loss or mutations. Nine TNBC patients were included in the cohort. The median PFS was only 8 weeks (95% CI: not specified), suggesting limited clinical efficacy in advanced breast cancer with PI3K/Akt/PTEN alterations (Table 1) [66].

### 5.3. mTOR Inhibitors in TNBC

Allosteric mTOR inhibitors (Figure 1), rapamycin and its analogues (everolimus and temsirolimus) specifically inhibit mTORC1 [67]. The single-arm phase II clinical trial NCT01127763 assessed the clinical benefit of everolimus in combination with carboplatin for metastatic TNBC. The median PFS for the 25 patients included in the study was 3 months (95% CI: 1.6–4.6 months), and the median OS was 16.6 months (95% CI: 7.3 months—not reached) (Table 1). The study reported frequent grade 3 or higher thrombocytopenia (28% of patients), mainly attributed to carboplatin, which was mitigated by lowering the dose of carboplatin [68]. Additionally, the phase I/II clinical trial NCT01939418 evaluated the combination of everolimus with cisplatin and gemcitabine. The median PFS was 5.5 months (95% CI: 3.5–7.5), and the median OS was 19.1 months (95% CI: 7.5–30.7). However, the study was terminated due to slow recruitment and an efficacy judged insufficient by the authors [69]. Last, in the phase II clinical trial NCT00930930, cisplatin/paclitaxel with everolimus did not improve pCR rates compared to placebo (36% versus 49%) [70].

ATP-competitive inhibitors (Figure 1) have also been developed, targeting both mTORC1 and mTORC2 [67], but few of them have yet been tested in clinical trials, notably in TNBC. One such inhibitor, TAK-228, associated with the PI3K-α inhibitor TAK-117, was tested in the pilot clinical trial NCT03193853, followed by cisplatin and nab-paclitaxel. Patients eligible for immunotherapy were then treated with pembrolizumab. The authors hypothesized that the use of TAK-228 and TAK-117 would increase DNA damage repair deficiency, leading to an improvement in sensitivity to DNA-damaging chemotherapy. Among the 10 patients included in the study, 1 showed a partial response, and 2 had stable disease ≥ 6 months after cisplatin + nab-paclitaxel. Interestingly, those three patients had PFS with pembrolizumab post-cisplatin/nab-paclitaxel of 1.2, 2 and 3.6 years, respectively (Table 1) [71].

### 5.4. Dual PI3K/mTOR Inhibitors in TNBC

Structural analyses of mTOR and PI3K revealed similarities in their catalytic enzymatic domains, leading to the development of dual PI3K/mTOR inhibitors in order to overcome resistance mechanisms mediated by compensatory feedback loops and pathway crosstalk [52]. One such dual inhibitor, XS-2, a thiophenetriazine derivative, showed anti-proliferative activity against TNBC cell line MDA-MB-231 (inhibition rate > 50% at 24, 48 and 72 h). Similarly, cytotoxicity assays demonstrated a half maximal inhibitory concentration (IC50) of 0.087 +/− 0.09 µM. Interestingly, XS-2 demonstrated low toxicity on normal cell LO-2 with an IC50 value > 100 µM. These preclinical results suggest that XS-2 may represents a candidate for development of low-toxicity therapeutic option in TNBC [72]. Similarly, samotolisib (LY3023414) (Figure 1) induced 77.6% tumor volume inhibition compared to control in the MDA-MB-231 orthotopic tumor model [73]. At the clinical stage, gedalotlisib (PF-05212384), a dual PI3K/mTOR inhibitor developed to reduce chemotherapy resistance, was tested in a cohort of patients including 22 patients with metastatic or locally recurrent/advanced TNBC in association with cisplatin in first-line (*n* = 10) or in second- or third-line therapy (*n* = 12) in the phase Ib trial NCT01920061. The median PFS in the first-line subgroup was 4.8 months (95% CI: 0.8–7.0) and 8.5 months (95% CI: 1.2—not evaluable) for the second- and third-line therapy subgroup, suggesting a good but limited efficacy in TNBC (Table 1) [74].

**Table 1:** PIK3/Akt/mTOR inhibitors used in clinical trial in triple-negative breast cancer (TNBC).

Inhibitor (Reference) *	Trial (NCT Identifier)	Study Phase	Main Results	Main Side Effects **
Buparlisib [55]	NCT01790932/NCT01629615	II	mPFS: 1.8 months.	Fatigue, nausea, hyperglycemia
Buparlisib [56]	NCT01572727	II/III	mPFS (buparlisib + paclitaxel vs. placebo + paclitaxel): 5.5 vs. 9.3 months.	Diarrhea, alopecia, rash
AZD8186 [58]	NCT01884285	I	No specific data on TNBC; 4/161 patients only experienced dose-limiting toxicities.	Diarrhea, nausea, fatigue
Alpelisib [59]	NCT02506556	II	mPFS: 1.8 months.	Hyperglycemia, rash, colitis
Taselisib [60]	NCT01296555	I	mPFS: 3.0 months.	Diarrhea, nausea, fatigue
Eganelisib [61]	NCT02637531	I/Ib	No antitumoral effect as monotherapy in TNBC.	Transaminase elevation, rash, fatigue
Capivasertib [63]	NCT02423603	II	mPFS in PIK3CA/Akt/PTEN altered subgroup (capivasertib + paclitaxel vs. placebo + paclitaxel): 9.3 vs. 3.7 months.	Diarrhea, fatigue, nausea
Ipatasertib [64]	NCT02162719	II	mPFS in PTEN-low subgroup (ipatasertib + paclitaxel vs. placebo + paclitaxel): 6.2 vs. 3.7 months.	Diarrhea, neutropenia
Ipatasertib [65]	NCT02301988	II	ORR improved by ipatasertib by 23.7% in PTEN-low population and by 14.9% in PIK3CA/Akt1/PTEN altered population vs. placebo.	Diarrhea, asthenia, peripheral neuropathy
MK-2206 [66]	NCT01277757	II	mPFS: 8 weeks.	Fatigue, rash, vomiting
Everolimus [68]	NCT01127763	II	mPFS (everolimus + carboplatin): 3 months.	Hematological toxicity, mucositis, dehydration
Everolimus [69]	NCT01939418	I/II	mPFS (everolimus + cisplatin + gemcitabine vs. cisplatin + gemcitabine): 5.5 vs. 5.7 months.	Neutropenia, stomatitis, Anorexia
Everolimus [70]	NCT00930930	II	pCR (everolimus + cisplatin + paclitaxel vs. cisplatin + paclitaxel + placebo): 36% vs. 49%.	Mucositis, transaminase elevation, rash
TAK-228 [71]	NCT03193853	II	*n* = 10 metastatic TNBC patients. Partial response: 1/10 patient. Stable disease ≥ 6 months: 2/10 patients after cisplatin and nab-paclitaxel.	Fatigue, nausea, diarrhea
Gedatolisib [74]	NCT01920061	Ib	mPFS in 1st-line subgroup: 4.8 months. mPFS in 2nd/3rd-line subgroup: 8.5 months.	Neutropenia, mucositis, alopecia

* PI3K/Akt/mTOR inhibitors have been classified according to their order of appearance in the manuscript (Section 5). ** For each clinical trial cited, adverse events are ranked by their frequency. Abbreviations: mPFS: median progression-free survival; ORR: overall response rate; pCR: pathological complete response; TNBC: triple-negative breast cancer; vs.: versus.

## 6. Resistance Mechanisms to Therapies Targeting the PI3K/Akt/mTOR Pathway

Various resistance mechanisms to therapies targeting the PI3K/Akt/mTOR pathway have been described, limiting their therapeutic efficacy in TNBC.

### 6.1. Resistance Mechanisms to PI3K Blockade

As far as PI3K inhibition is concerned, numerous mechanisms of drug resistance have also been described.

First of all, due to the involvement of the PI3K/Akt pathway in glycolysis [1], PI3K inhibition induces hyperglycemia, which activates insulin signaling, especially through the insulin-like growth factor 1 receptor (IGF1R). This signaling can in turn reactivate the PI3K/Akt/mTOR pathway [75], activate the Janus kinase 2 (JAK2)/signal transducer and activator of the transcription 5 (STAT5) pathway to promote cell proliferation and survival [76] or activate the oncogenic kinase MAPK4, which can activate the Akt/mTOR axis independently of PI3K, conferring resistance to PI3K inhibitors, particularly in BL subtypes where MAPK4 is often overexpressed [77].

In parallel, alternative mTOR activation pathways, such as PIM1 kinase overexpression, can maintain mTOR activity despite PI3K inhibition [49].

Moreover, overexpression of the pseudo RTK protein kinase 7 (PTK7), frequently observed in TNBC, can lead to a quick activation of the non-canonical Wingless-related integration (Wnt) pathway upon PI3K inhibition [78]. Additionally, the feedback loop between PI3Kα and β isoforms can restore pathway activity when isoform-selective inhibitors are used [57].

### 6.2. Resistance Mechanisms to mTOR Blockade

Everolimus-induced blockade of the mTOR pathway leads to compensatory activation of the mitogen-activated protein kinase (MAPK) pathway through increased interaction between c-Rapidly accelerated fibrosarcoma (Raf) and SHOC2 [79]. Activation of the MAPK pathway is thus associated with high virulence of TNBC and, in particular, with the metastatic process [80].

Moreover, treatment with everolimus tends to increase Akt phosphorylation through a feedback loop triggered by mTORC2 [81].

Resistance to everolimus monotherapy may also be due to reduced T cell-mediated immunity. In fact, mTOR inhibition increases PD-L1 expression on tumor endothelial cells, facilitating cancer immune escape [82].

In addition, mTORC1 inhibition may activate the focal adhesion kinase (FAK) pathway, compensating for the downregulation of extracellular matrix-related gene expression of TNC, collagen type VII alpha 1 chain (COL7A1) and collagen type VIII alpha 2 chain (COL8A2) [83].

### 6.3. Other Resistance Mechanisms

AXL receptors, members of the TAM family of RTKs, are often overexpressed in M and MSL subtypes. They are activated by phosphatidylserine found on apoptotic bodies, thereby promoting pro-tumorigenic signaling and drug resistance. Their overexpression is associated with a poor prognosis [84].

Finally, hyperactivation of autophagy represents another resistance mechanism, particularly in response to PI3K and Akt inhibitors [85]. Since autophagy is negatively regulated by mTORC1, its inhibition leads to autophagy activation, which increases drug resistance in TNBC by enhancing cellular tolerance to hypoxia [32].

## 7. Targeted Therapies Combined with PI3K/Akt/mTOR Pathway Inhibitors

The exploration of resistance mechanisms to therapies targeting the PI3K/Akt/mTOR pathway has led to the development of combination strategies with other targeted agents to enhance their efficacy. In addition, increasing knowledge of the genetic and mutational profiles that can lead to drug resistance raises the question of the urgent need for diagnostic methods to propose the most appropriate targeted therapies to patients.

### 7.1. Combination with Antibody–Drug Conjugate (ADC)

The combination of gedatolisib with the ADC cofetuzumab pelidotin, targeting PTK7, was evaluated in the phase I clinical trial NCT03243331. This combination of treatments aimed at circumventing the resistance mechanism driven by the Wnt pathway activation through PTK7 inhibition. Moreover, the auristatin payload of cofetuzumab pelidotin demonstrated synergistic activity with gedatolisib in preclinical studies, providing a strong rationale for the combination. In this trial, the median PFS was 2 months (95% CI: 1.2–6.2) (Table 2). Regarding safety, 5 out of 18 patients included in the study experienced grade 3 or higher toxicity (nausea: *n* = 1, fatigue: *n* = 2, myelosuppression: *n* = 2), suggesting an acceptable toxicity profile [78].

### 7.2. Combination with Poly (ADP-Ribose) Polymerase (PARP) Inhibitors

Preclinical studies have demonstrated a synergistic potential between PI3K inhibitors and PARP inhibitors. PI3K inhibition reduces BRCA1 expression and impairs nucleotide synthesis, both necessary for DNA repair. Based on these findings, a phase I study (NCT01623349) was conducted in 26 BC patients, including 14 TNBC, to evaluate the combination of the PI3K inhibitor buparlisib and the PARP inhibitor olaparib. Among BC patients, five (28%) showed partial response, and eight (44%) achieved a stable disease for over 6 months, suggesting promising results for this therapeutic association [86]. Likewise, the phase I clinical trial NCT04586335 evaluating the PI3K inhibitor CYH33 and olaparib was conducted, but results have not yet been reported (Table 2) [87].

### 7.3. Combination with Eribulin

As previously discussed, preclinical studies have demonstrated a synergistic association between eribulin and everolimus or PI3K inhibitor copanlisib in TNBC cell lines [53]. These findings led to the phase I study NCT02120469, evaluating eribulin combined with everolimus, which showed a median PFS of 2.6 months (95% CI: 2.1–4.0), suggesting modest efficacy [88]. The phase I/II trial NCT04345913, evaluating the combination of copanlisib and eribulin in patients with metastatic TNBC is currently under investigation, and, to our knowledge, no results have yet been reported (Table 2) [89].

### 7.4. Combination with Nab-Paclitaxel +/− Immunotherapy

The phase Ib study NCT03800836 evaluated the efficacy and safety of ipatasertib/atezolizumab/paclitaxel or nab-paclitaxel in patients with locally advanced or metastatic TNBC (*n* = 114). The rationale for combining ipatasertib with an anti-PD-L1 agent is that AKT inhibitors may restore T-cell functions in the tumor microenvironment and promote the expansion of memory cells with a stem cell-like phenotype. Ipatasertib could, therefore, enhance checkpoint inhibitor efficacy by maintaining a stem-like phenotype in memory T cells, thereby promoting a long-term response in patients. The median PFS was similar when patients received nab-paclitaxel (median PFS 6.6 months (95% CI: 3.4–9.2)) or paclitaxel (median PFS 7.2 months (95% CI: 5.3–7.4)). Subgroup analysis according to PIK3CA/Akt/PTEN alteration status showed neither no clear difference: the median PFS was 7.4 months in patients with PIK3CA/Akt/PTEN alterations and 6.6 months in patients without such alterations. The most common grade 3 or 4 adverse events were diarrhea (12%), neutropenia (11%) and rash (10%). Two fatal adverse events occurred during the study (pneumonia and ischemic stroke) [90].

Likewise, the EPIK-B3 phase III CT evaluating the alpelisib/nab-paclitaxel combination in patients with advanced TNBC with PIK3CA mutation or PTEN loss is ongoing. This study should make it possible to optimize this therapeutic combination according to the molecular profile of TNBC patients. The results of this trial are awaited with interest in the context of personalized medicine [91].

The association eganelisib/atezolizumab/nab-paclitaxel was evaluated in the inoperable locally advanced or metastatic TNBC subgroup in the single-arm MARIO-3 phase II trial. Preliminary efficacy data showed a median PFS of 11.0 months in the PD-L1 positive subgroup (*n* = 14) and 7.3 months in the PD-L1 negative subgroup (*n* = 27) (Table 2) [92].

### 7.5. Combination with Androgen Receptor (AR)-Inhibitor

Preclinical data have suggested higher sensitivity of AR-positive TNBC to PI3K inhibitors and AR antagonists. These findings led to the development of the phase Ib/II TBCR032 trial (NCT02457910), comparing the effectiveness of enzalutamide alone or combined with taselisib 4 mg in AR-positive TNBC patients. The median PFS was better in the LAR subtype (4.6 versus 2.0 months, *p* = 0.082), supporting a potential benefit of this strategy in this molecular subgroup (Table 2) [93].

### 7.6. Diagnostic Methods to Optimize Targeted Therapy Utilization

Although still relatively poorly understood, the mutational profile of the PI3K/Akt/mTOR pathway could play an important role in the efficacy of targeted and combination therapies in TNBC. For example, PI3KCA-mutated TNBC patients may be more sensitive to anti-androgen therapies than other TNBC patients [93]. Similarly, as mentioned above, the importance of PTEN in overactivating the signaling pathway is gradually leading to the development of clinical trials such as EPIC-B3, aimed at improving disease prognosis according to the PTEN profile [91].

In this context, there is an urgent need for the development of diagnostic methods to identify the mutation profile of TNBC patients. For example, to identify PIK3CA mutations, RT-PCR methods have been developed to detect the main mutations described [94]. In addition, although more complex, the detection of PTEN loss is also possible by immunohistochemistry or the detection of copy-number variation by next-generation sequencing [15].

These methods, coupled with clinical trials enabling more precise assessment of treatment response according to TNBC mutational profile, could improve patient care.

However, the very high heterogeneity of TNBC and its numerous resistance mechanisms remain a limitation to the efficacy of targeted therapies.

## 8. Conclusions and Future Directions

The PI3K/Akt/mTOR pathway is a major tumor signaling pathway, implicated in numerous oncogenic processes, including cell proliferation and migration and inhibition of apoptosis. It plays also a central role in chemoresistance, notably to conventional cytotoxic therapies. Particularly, this pathway is hyperactivated in the majority of TNBC cases, essentially caused by activating mutations in *PIKCA* and *AKT1* and loss-of-function alterations in PTEN. These alterations contribute to aggressive cancer phenotypes and correlate with poor clinical outcomes. A considerable research effort has, therefore, been made to identify these mutations and establish genetic profiles to predict disease severity and response to treatment. Technologies such as RT-PCR, immunohistochemistry and next-generation sequencing are increasingly being used to determine the mutational profiles of patients. These developments should be pursued with a view to integrating them into the biological diagnosis of patients with TNBC.

This makes the PIK3/Akt/mTOR pathway a prime target for the development of targeted therapies. As a result, numerous PI3K, Akt, mTOR and dual PI3K/mTOR inhibitors have been developed. However, despite promising preclinical results, they harbored a high toxicity-profile-complicating patient adherence. In addition, due to the very high molecular heterogeneity of TNBC, several molecular resistance mechanisms have emerged, involving compensatory signaling pathways such as MAPK or Wnt. These factors contribute to their limited clinical efficacy to date.

To gain a better understanding of this signaling pathway and its heterogeneity, recent techniques are progressively emerging. These include organoids, which provide a better view on the cellular impact of the activation of this signaling pathway. Another is spatial transcriptomics, which could enable us to identify the specific cellular sites of activation of the elements described during cancerous progression.

Nonetheless, recent advances in molecular characterization and diagnostic tools in cancer are gradually leading to the development of combined treatment strategies, associating PI3K/Akt/mTOR inhibitors with therapies targeting compensatory signaling pathways. The first results of these trials seem promising, particularly in TNBC patients with PTEN loss-of-function alterations. Although further studies and a deeper understanding of TNBC molecular mechanisms are still required, these results raise hopes for improving clinical outcomes in this disease in the era of personalized medicine. This is encouraging researchers and clinicians to develop biomarker-driven clinical trials adapted to new biological knowledge and therapeutic advances.

## Figures and Tables

**Table 2:** Clinical trial of PI3K/Akt/mTOR inhibitors used in combination with other targeted therapies in triple-negative breast cancer (TNBC).

Combined Therapy	Trial (NCT Identifier)	Phase	Drug	Patients	Treatment	Reference
Association with Antibody Drug Conjugate	NCT03243331	I	Gedatolisib	Metastatic TNBC	Gedatolisib plus Cofetuzumab pelidotin	[78]
Association with PARP inhibitor	NCT01623349	I	Buparlisib	High-grade serous ovarian cancer and TNBC	Buparlisib plus olaparib	[86]
	NCT04586335	I	CYPH33	Advanced solid tumors	CYH33 plus olaparib	[87]
Association with eribulin	NCT02120469	I	Everolimus	Metastatic TNBC	Everolimus plus eribulin	[88]
	NCT04345913	I/II	Copanlisib	Advanced-stage TNBC	Copanlisib plus eribulin	[89]
Association with nab-paclitaxel +/− immunotherapy	NCT03800836	Ib	Ipatasertib	Locally advanced or metastatic TNBC	Ipatasertib plus atezolizumab plus paclitaxel or nab-paclitaxel	[90]
	NCT04251533	III	Alpelisib	Advanced TNBC	Alpelisib plus nab-paclitaxel	[91]
	NCT03961698	II	Eganelesib	Locally advanced and/or metastatic TNBC or Renal cell carcinoma	Eganelisib plus atezolizumab plus nab-paclitaxel	[92]
Association with Androgen Receptor inhibitor	NCT02457910	Ib/II	Taselisib	Androgen receptor positive TNBC	Taselisib plus enzalutamide	[93]
